# Curative resection after chemotherapy and chemoradiotherapy for postoperative recurrence of pancreatic tail cancer in the abdominal wall: a case report

**DOI:** 10.1186/s40792-022-01452-3

**Published:** 2022-05-19

**Authors:** Shunya Iio, Yuto Hozaka, Kiyonori Tanoue, Tetsuya Idichi, Kousuke Fukuda, Taiki Nakashima, Ryutaro Yasudome, Yoichi Yamasaki, Yota Kawasaki, Takaaki Arigami, Akihiro Nakajo, Michiyo Higashi, Yuko Mataki, Hiroshi Kurahara, Takao Ohtsuka

**Affiliations:** 1 Department of Digestive Surgery, Breast and Thyroid Surgery, Graduate School of Medical and Dental Sciences, Kagoshima University, 8-35-1, Sakuragaoka, Kagoshima, 890-8520 Japan; 2 Department of Pathology, Graduate School of Medical and Dental Sciences, Kagoshima University, 8-35-1 Sakuragaoka, Kagoshima, 890-8544 Japan

**Keywords:** Pancreatic ductal adenocarcinoma, Abdominal wall recurrence, Multidisciplinary therapy, Surgical margin, Femoral myocutaneous flap

## Abstract

**Background:**

Locoregional recurrence and metastasis to the liver, peritoneum, and lung are the most common recurrent patterns of pancreatic ductal adenocarcinoma (PDAC) after radical resection. Recurrence in the abdominal wall is extremely rare. Herein, we report our experience with a patient who had recurrent PDAC in the abdominal wall with long-term survival by means of multidisciplinary therapy.

**Case presentation:**

A 76-year-old Japanese woman was diagnosed with resectable pancreatic tail cancer. She underwent distal pancreatectomy with regional lymphadenectomy after two cycles of gemcitabine plus S-1 as neoadjuvant therapy. She also received eight cycles of S-1 as adjuvant chemotherapy. Approximately 14 months after the initial surgery, imaging examinations identified a mass suggesting recurrence in the abdominal wall at the middle wound that involved the transverse colon. After two cycles of gemcitabine plus nab-paclitaxel, chemoradiotherapy (S-1 plus 45 Gy) and seven cycles of modified FOLFIRINOX (5-fluorouracil/leucovorin, irinotecan, and oxaliplatin) were administered. The patient did not develop any new recurrent lesions during chemotherapy and chemoradiotherapy. Therefore, the recurrent lesion in the abdominal wall and the involved transverse colon were resected. We confirmed the lack of peritoneal dissemination during surgery. Pathological examination revealed that the resected lesion was metastasis of primary PDAC, and the surgical margin was 1 mm. However, re-recurrence localized in the abdominal wall was detected 9 months later. The re-recurrent lesion was diagnosed as local recurrence of the first recurrent lesion. We performed a second resection of the abdominal wall using a femoral myocutaneous flap to achieve sufficient surgical margin. The pathological findings of the resected specimen were the same as those of the previous specimens, and the resection margin was negative. The patient’s postoperative course was uneventful. Seven years after the initial surgery and 3 years and 7 months after the third surgery, the patient is alive with no signs of recurrence.

**Conclusions:**

Long-term survival could be achieved by radical resection with sufficient surgical margins for recurrence of PDAC in the abdominal wall if new other recurrent lesions, including peritoneal dissemination, are prevented through chemotherapy.

## Background

Pancreatic ductal adenocarcinoma (PDAC) is becoming more common and is associated with a very poor prognosis [1, 2]. Even after radical resection, patients often experience locoregional recurrence, peritoneal dissemination, and distant organ metastasis. Disease control after recurrence is difficult. However, recent advances in multidisciplinary therapy have improved the overall survival of patients with advanced PDAC [2–4]. Several studies have reported that multidisciplinary therapy controls recurrent PDAC lesions and contributes to long-term survival [4–6]. Herein, we report a case of PDAC with postoperative abdominal wall recurrence with long-term survival using multidisciplinary therapy.

## Case presentation

**Fig. 1:**
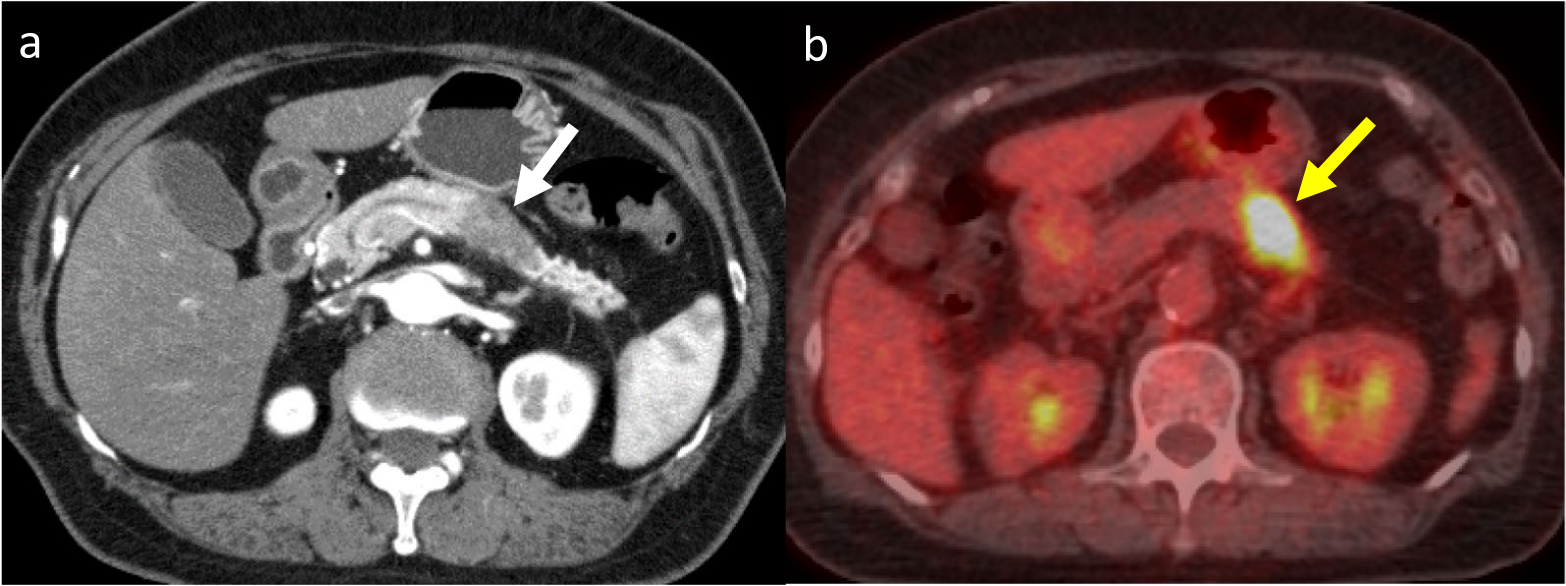
Imaging results at admission. **a** Contrast-enhanced abdominal computed tomography showed a 35-mm hypovascular mass in the tail of the pancreas (white arrow). **b** ^18^F-fluorodeoxyglucose-positron-emission tomography/computed tomography showed high accumulation of fluorodeoxyglucose in the tumor (yellow arrow)

A 76-year-old Japanese woman was admitted to our hospital because she was incidentally found to have a pancreatic tumor during a regular health checkup. The patient did not have any family members with pancreatic cancer. Her serum carbohydrate antigen 19-9 (CA19-9) level was 217.0 U/mL (normal range, 0–37 U/mL). Other laboratory test results, including serum carcinoembryonic antigen level, were normal. Abdominal enhanced computed tomography (CT) showed a 35-mm hypovascular mass located in the pancreatic tail. The tumor involved the splenic artery and veins (Fig. 1a). Endoscopic ultrasound-guided fine-needle aspiration revealed a tubular adenocarcinoma. ^18^F-fluorodeoxyglucose (FDG)-positron-emission tomography (PET)/CT showed high FDG accumulation in the pancreatic tumor and no other abnormal accumulation suggesting distant metastasis or other solid tumors (Fig. 1b). Based on these findings, the patient was diagnosed with resectable PDAC (T2N0M0 stage IB) according to the 8th edition of the Union for International Cancer Control staging system. She was administered two cycles of neoadjuvant gemcitabine (1000 mg/m^2^, days 1 and 8 of a 21-day cycle) plus S-1 (100 mg/body on days 1 through 14 of a 21-day cycle), after which the tumor size decreased slightly from 35 to 32 mm, and her serum CA19-9 levels decreased to 75.3 U/mL. No serious adverse events were observed during chemotherapy.

She underwent distal pancreatectomy with regional lymphadenectomy after confirmation of no atypical cells on lavage ascites cytopathology. The midline wound was protected using a wound protector during the surgical procedure. The resected specimen was a whitish tumor 3.3 × 2.8 × 2.0 cm in size. Pathological examination revealed that the cancer cells were almost viable and had invaded the splenic vessels. The surgical margins were negative. The pathological stage was IIB (pT2N1M0). The patient’s postoperative course was uneventful. She received eight cycles of adjuvant S-1 (100 mg/body on days 1 through 14 of a 21-day cycle).

**Fig. 2:**
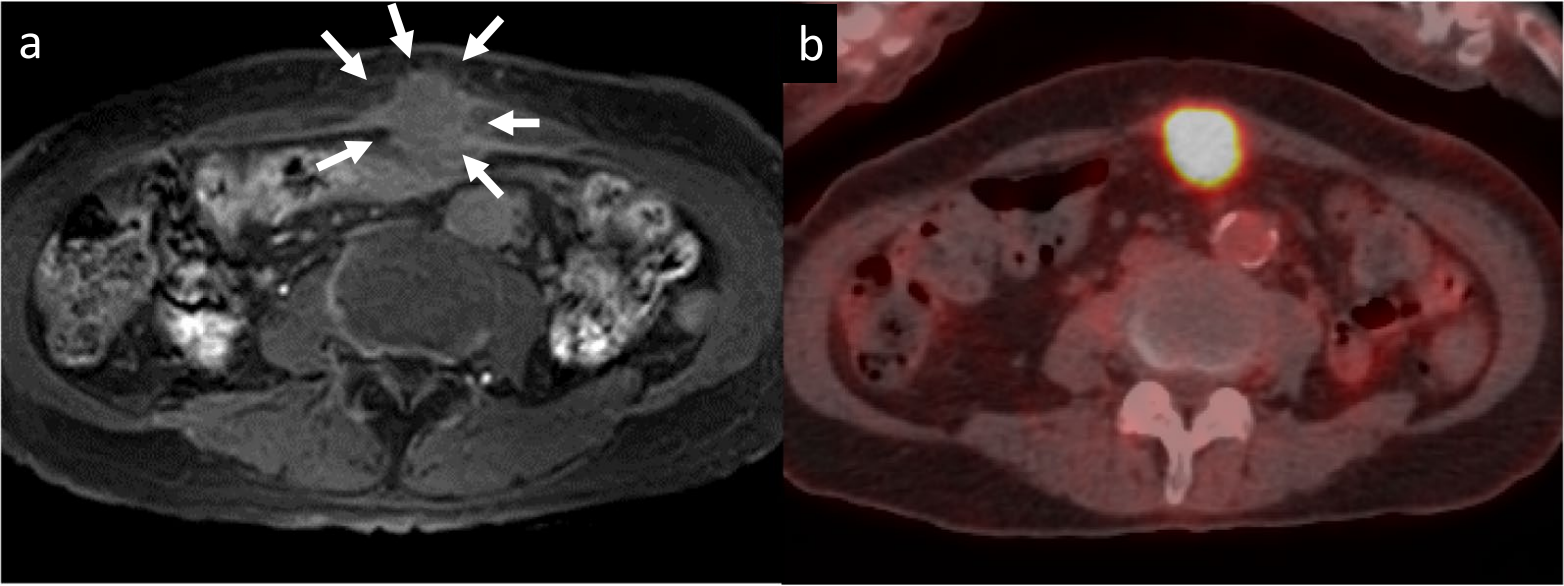
Imaging results 14 months after the initial surgery. **a** Contrast-enhanced abdominal magnetic resonance imaging showed a 30-mm low-intensity signal from the wound to the transverse colon in T1-weighted imaging that was suggestive of recurrence (white arrows). **b** ^18^F-fluorodeoxyglucose-positron-emission tomography/computed tomography showed high fluorodeoxyglucose accumulation in the tumor at the wound of the pancreatectomy

**Fig. 3:**
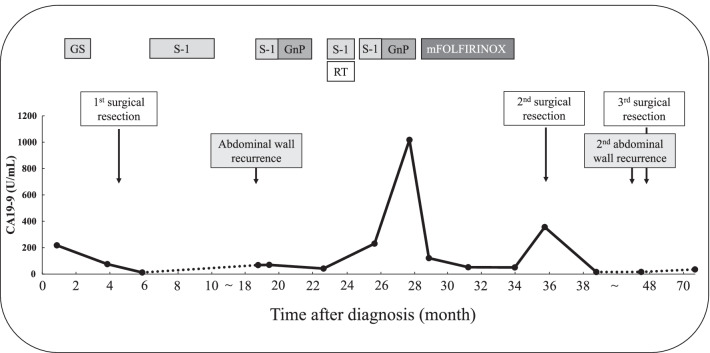
Clinical course and level of serum carbohydrate antigen 19-9. GEM gemcitabine, RT radiotherapy, GnP gemcitabine plus nab-paclitaxel therapy, mFOLFIRINOX modified 5-fluorouracil/leucovorin, irinotecan, and oxaliplatin

**Fig. 4:**
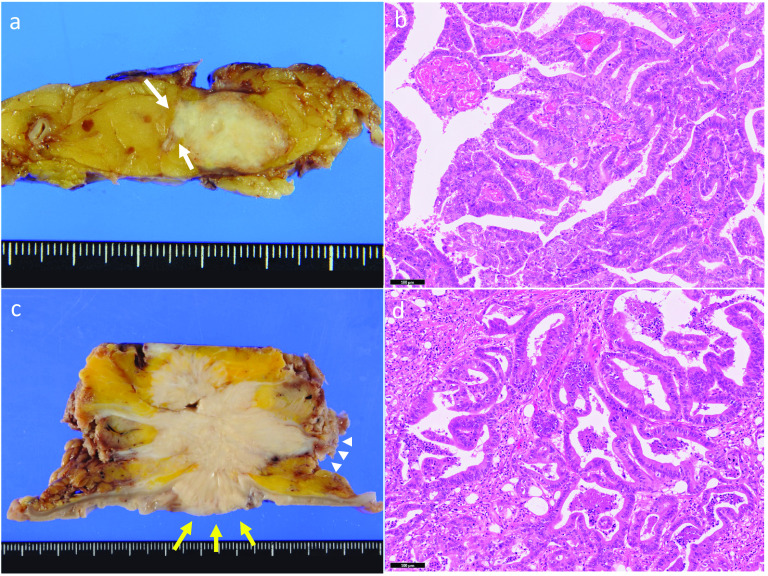
Gross and histopathological findings of the primary pancreatic cancer and abdominal wall recurrence. **a** The cut surface of the original resected specimen was 3.3 × 2.8 × 2.0 cm in size. The tumor was whitish and had invaded the splenic vessels (white arrows). **b** Microscopic findings (hematoxylin and eosin [HE] staining × 100) showed proliferation of the tumor cells, which were composed of large, atypical tubules, indicating a well-differentiated adenocarcinoma. **c** The cut surface of the resected first recurrent specimen was 6.0 × 4.8 × 4.5 cm in size. There were no tumor cells on the surgical margin, but it was close to the colonic mucosa (yellow arrows) and the transected plane (white arrowheads). **d** Microscopic findings (HE staining × 100) showed atypical ductal cells resembling primary pancreatic ductal adenocarcinoma

Approximately 14 months after surgery, abdominal plane CT and contrast-enhanced magnetic resonance imaging (MRI) showed a 30-mm mass in the abdominal wall at the middle wound that involved the transverse colon, suggesting postoperative recurrence (Fig. 2a). CT was performed without contrast medium because the patient experienced allergic symptoms on a previous CT with an iodine contrast medium. FDG-PET/CT showed high FDG accumulation in the abdominal wall lesion without abnormal accumulation in other organs (Fig. 2b). An endoscopic gastrointestinal examination revealed no malignant tumors. She was diagnosed with postoperative recurrence of PDAC. Regarding the recurrence pattern, it was difficult to distinguish abdominal wall infiltration of peritoneal dissemination from implantation in the abdominal wall. Considering the risk of rush progression from potential peritoneal dissemination, two cycles of GnP (gemcitabine 1000 mg/m^2^ and nab-paclitaxel 125 mg/m^2^ on days 1, 8, and 15 of a 28-day cycle) were administered. During chemotherapy, the tumor size decreased slightly, and no other distant metastases appeared. As we expected the resection range to reduce and surgical margins to be negative owing to tumor downsizing, we added chemoradiotherapy (45 Gy in 4 weeks with S-1 at a dose of 100 mg/body for the first 21 days). Subsequently, two cycles of GnP were administered as maintenance therapy, but the tumor slightly increased in size. Therefore, modified FOLFIRINOX (oxaliplatin, 85 mg/m^2^; irinotecan, 150 mg/m^2^; leucovorin, 400 mg/m^2^; and 5-fluorouracil, 2400 mg/m^2^) was administered (Fig. 3). After seven cycles of modified FOLFIRINOX, the tumor size decreased to 25 mm, and no other metastatic lesions had appeared. We performed surgical resection of the recurrent lesion. After intraoperative confirmation of no peritoneal dissemination and no obvious malignant cells by lavage ascites cytopathology, partial resection of the abdominal wall and transverse colon was performed. The resected specimen was a whitish tumor 6.0 × 4.8 × 4.5 cm in size, and pathological examination revealed it to be metastatic adenocarcinoma, consistent with a metastasis of the primary PDAC (Fig. 4). The surgical margin was 1 mm. The patient’s postoperative course was uneventful.

**Fig. 5:**
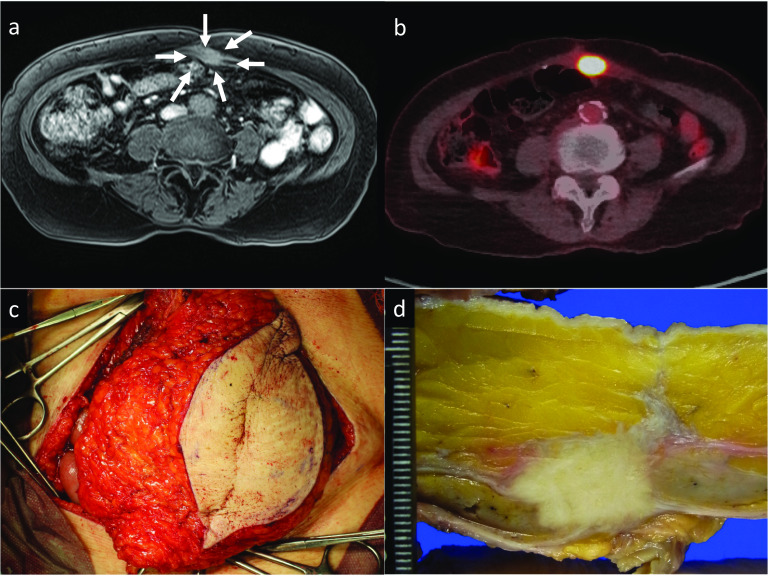
Imaging and intraoperative and gross findings of the second abdominal wall recurrence. **a** Contrast-enhanced abdominal magnetic resonance imaging showed a 15-mm iso-intensity signal localized in the abdominal wall in T1-weighted imaging that was suggestive of recurrence (white arrows). **b** ^18^F-fluorodeoxyglucose-positron-emission tomography/computed tomography showed high fluorodeoxyglucose accumulation in the mass in the abdominal wall. **c** Intraoperative findings. The recurrent lesion was resected with a sufficient margin, and the abdominal wall was repaired using a femoral myocutaneous flap. **d** The cut surface of the resected specimen showed no tumor cells in the transected plane or the intra-abdominal plane

Nine months after resection of the recurrent lesion, abdominal contrast-enhanced MRI showed a new mass in the abdominal wall at the site of the previous wound (Fig. 5a). FDG-PET/CT also showed abnormal FDG accumulation in the tumor (Fig. 5b). We diagnosed the lesion as re-recurrence from the first recurrent lesion due to insufficient surgical margins. As the mass was localized within the abdominal wall, we performed resection of the re-recurrent lesion with sufficient surgical margins (Fig. 5c). We repaired the large defect of the abdominal wall after resection using a femoral myocutaneous flap. The pathological findings of the resected specimen were the same as those of the previous specimens (Fig. 5d). The surgical margin was 20 mm. The patient’s postoperative course was uneventful. Seven years after the initial surgery and 3 years and 7 months after the third surgery, the patient is alive without recurrence.

## Discussion

Recurrence of PDAC often appears within 2 years after surgery and frequently occurs in the liver, peritoneum, locoregional organs, or lung [7–9]. Postoperative recurrence of PDAC in the abdominal wall is extremely rare, and its characteristics are not well known. We experienced a recurrence in the abdominal wall that was treated with multidisciplinary therapy.

We searched the PubMed database for studies published in English using the terms “abdominal wall recurrence” and “pancreatic cancer” or “pancreatic ductal adenocarcinoma”. There may be publication bias, but to the best of our knowledge, there have been only two previous reports of pancreatic cancer with postoperative abdominal wall recurrence [10, 11]. Billesbolle et al. reported their experience with a 62-year-old man with a single recurrence within the abdominal wall 4 months after distal pancreatectomy for pancreatic tail cancer [10]. After resection of the abdominal wall recurrence, local recurrence at the gastric wall that occurred 10 months later was also resected. He survived for 6 years after the third surgery. Young et al. reported their experience with a 57-year-old woman with abdominal wall recurrence after laparoscopic pancreaticoduodenectomy for pancreatic head cancer [11]. She had simultaneous recurrences at the three port-sites of the abdominal wall soon after adjuvant chemoradiotherapy and died 15 months after surgery. Recurrence in the abdominal wall appeared in all three cases, including the present case, at the site of the surgical wound within 2 years after the initial pancreatectomy. However, our case is the only one that was controlled with multidisciplinary therapy including systemic chemotherapy, chemoradiotherapy, and surgical resection.

Most metastatic and recurrent PDACs develop multiple tumors and become uncontrolled in a short period of time, requiring a shift in treatment from chemotherapy to best supportive care. However, there are rare cases of sporadic recurrence or oligo-metastases that can be controlled by multidisciplinary therapy that combines multidrug chemotherapy and radiotherapy [2, 5, 6]. Lung metastases that occur late after surgery may remain oligometastatic, and the prognosis is better than that of other metastatic recurrences [12–14]. Two of the three patients with abdominal wall recurrence after pancreatectomy for PDAC had localized and resectable disease, indicating that it may be a controllable recurrence. It is difficult to determine the optimal timing for resection of a recurrent lesion. If the recurrent lesion is localized within the abdominal wall owing to implantation, curative resection may improve the prognosis. Considering that no other type of recurrence such as peritoneal dissemination and distant metastasis occurred, implantation of cancer cells might have caused the first recurrence in the abdominal wall in the present case. The risk of abdominal wall recurrence due to implantation is based on four factors: (1) biological invasiveness of the primary tumor; (2) local traumatic factors; (3) host immune response, and (4) surgical technique [15, 16]. Even if we perform proper intraoperative management (wound protection and minimization of tumor treatment), it may be difficult to prevent implantation-induced recurrence completely. Because it was difficult to distinguish abdominal wall infiltration of peritoneal dissemination from implantation in the abdominal wall at the time of diagnosis, we performed systemic chemotherapy considering a risk of rush progression with peritoneal dissemination or appearance of other distant metastases. Furthermore, we administered chemoradiotherapy to reduce the tumor size, but the recurrent lesion did not shrink. Therefore, chemoradiotherapy did not have a therapeutic effect in the present case.

After resection of the first recurrent lesion, pathological examination revealed the surgical margin of 1 mm and the second recurrence occurred at the same site. It is presumed that cancer cells invading beyond resection line were left between the thin sections that were evaluated every 5 mm. Since insufficient surgical margins might cause the second recurrence, we resected the lesion with sufficient surgical margins and used a femoral myocutaneous flap. Although the necessity and duration of chemotherapy before surgical resection for recurrent lesions in the abdominal wall that involve intraabdominal organs are unclear, complete resection with sufficient surgical margins could lead to long-term survival if peritoneal dissemination or other distant metastasis do not appear during the chemotherapy. Accumulation and analysis of clinical characteristics and therapeutic effects are important to establish the optimal therapeutic strategy for rare presentations, such as PDAC recurrence in the abdominal wall.

## Conclusions

Long-term survival may be achieved with radical resection with sufficient surgical margins for recurrence of PDAC in the abdominal wall when it is sporadic and can be controlled with chemotherapy.

## Data Availability

Not applicable.

## Abbreviations

PDAC: Pancreatic ductal adenocarcinoma
CA19-9: Carbohydrate antigen 19-9
CT: Computed tomography
FDG: 18^F^-Fluorodeoxyglucose
PET/CT: Positron-emission tomography/computed tomography
MRI: Magnetic resonance imaging
GnP: GEM plus nab-paclitaxel therapy
FOLFIRINOX: 5-Fluorouracil/leucovorin, irinotecan, and oxaliplatin therapy
